# Exploring the Role of Footwear in Trail Running and Hiking: A Scoping Review

**DOI:** 10.1002/jfa2.70197

**Published:** 2026-08-08

**Authors:** Aaron Jackson, Kiseon Hong, Mike Frecklington, Kelly Sheerin, Matthew Carroll

**Affiliations:** ^1^ School of Allied Health Faculty of Health & Environmental Sciences Auckland University of Technology Auckland New Zealand; ^2^ Sports Performance Research Institute New Zealand (SPRINZ) Faculty of Health & Environmental Sciences Auckland University of Technology Auckland New Zealand; ^3^ Active Living and Rehabilitation: Aotearoa New Zealand Health and Rehabilitation Research Institute School of Clinical Sciences Auckland University of Technology Auckland New Zealand

**Keywords:** biomechanics, comfort, injury, off‐road, performance, physiology, shoes

## Abstract

**Introduction:**

Off‐road activities such as trail running and hiking have undergone a significant surge in global popularity over the last 2 decades, but this engagement also raises the potential for musculoskeletal injuries, which are prevalent and comparable to rates reported in road running. Footwear is critical in these dynamic environments, incorporating features such as aggressive outsole patterns and structural stability elements to accommodate varied and rugged terrain.

**Methods:**

Given the limited synthesis and fragmented nature of the literature, this scoping review systematically mapped and summarised existing research on the influence of footwear in hiking and trail running. The review was guided by three research questions that examined key outcome domains and methodological approaches, using the Arksey and O'Malley framework, and was reported in accordance with PRISMA‐ScR guidelines.

**Results:**

A search across four databases identified 16 eligible studies. Thirteen studies (81%) focused on trail running, exploring various footwear types, including advanced footwear technology, minimalist designs, and specific midsole characteristics. Biomechanical outcomes were the most frequently investigated (62% of studies), while physiological outcomes, performance, injury, and comfort were addressed in 25%–31% of studies each.

**Conclusion:**

Although methodological strengths include an increasing commitment to ecological validity by leveraging wearable sensors in combined lab and field protocols, persistent limitations include frequent use of small sample sizes and the underrepresentation of female participants in many controlled trials. A significant evidence gap remains regarding the chronic effects of prolonged footwear use and the interaction of footwear with fatigue. Future research requires methodological standardisation and longitudinal studies to advance toward individualised, evidence‐based footwear solutions.

## 1 Introduction

Off‐road activities such as trail running and hiking have undergone a surge in global popularity over the last 2 decades [1, 2, 3]. Notably, trail running alone has seen a 15% increase in participation in the past 10 years [4], while long‐distance hiking, exemplified by journeys along trails like the Appalachian and Pacific Crest, continues to attract a growing number of outdoor enthusiasts [5]. These activities are uniquely defined by natural environments that feature varied and rugged terrain, significant elevation changes, and natural obstacles such as rocks and roots [4, 6, 7]. In contrast to the predictable, flat surfaces typical of road running or walking, these dynamic conditions require heightened adaptability [5, 6] and elicit distinct physiological and biomechanical responses [8].

Musculoskeletal injuries are prevalent in trail running [9]. Hamill et al. [6] surveyed over 1000 off‐road runners and reported an injury prevalence of almost 40% within the preceding 12 months, a rate comparable to road running [10]. Trail running injuries are more frequently associated with uneven terrain, with acute ankle sprains the most commonly reported [6]. Other injuries include heel pain, fractures across various anatomical sites, knee pain, and shin pain [6]. Paraesthesia, blisters, and falls are among the most frequently reported issues encountered during hiking [5, 11, 12]. However, the overall evidence base regarding injury rates and risk factors in trail running and hiking is limited to a small number of studies. Chrusch et al. [13] surveyed 1259 individuals who had completed or attempted the 3500 km Appalachian Trail in the eastern United States, finding that 61% of respondents experienced musculoskeletal complaints. Notably, 11% of respondents reported symptoms severe enough to discontinue their hike.

Footwear designed for use in off‐road environments has purposeful features that differentiate it from road running shoes. It has been reported that runners seek traction, cushioning, and protection when selecting trail running footwear [6]. In contrast to road running shoes, which are typically optimised for predictable surfaces, lightweight construction, and energy return, off‐road footwear must accommodate irregular terrain, variable environmental conditions, and different mechanical demands. As such, trail running and hiking footwear often incorporates aggressive outsole lug patterns for grip, reinforced uppers for durability and protection, and structural elements that enhance stability on uneven ground. These design features reflect the distinct biomechanical and environmental challenges encountered in off‐road activities.

Along with growing participation in trail running, there is an increasing interest in running further and faster [14]. The physiological and biomechanical demands associated with trail running differ from those linked to road running [8]. Nevertheless, current biomechanical and performance frameworks remain largely road‐centric, primarily due to the limited availability of trail‐specific evidence and alternative concepts [15]. From a performance footwear perspective, carbon‐plated footwear, otherwise known as advanced footwear technology (AFT), has received the greatest research and public attention for its suggested performance benefits [4, 15]. In some studies, ATF footwear has been shown to improve running economy (RE) on the road by an average of 4%, and reduce race times at an elite level by 1%–2% [16, 17, 18]. Due to the performance focus of AFT, studies have generally assessed RE at speeds of 16–18 km/h [18]. However, the applicability of these speeds to trail running is limited, as even elite athletes tend to move considerably slower on sections of technical mountainous terrain [15].

Given the increasing popularity of hiking and trail running, and the critical role footwear plays in these activities, a comprehensive understanding of how footwear influences biomechanics, performance, physiology, comfort, and injury risk is essential. To address this need, a scoping review was conducted to systematically map existing research on the influence of footwear in hiking and trail running. This review aimed to clarify current knowledge, identify key themes, and highlight areas for future investigation. Three primary research questions guided the development of this study: (1) What biomechanical, physiological, and performance‐related outcomes have been investigated in relation to trail running and hiking footwear? (2) How have injury and comfort been defined and examined in the context of trail running and hiking footwear, and what approaches have been used to assess these outcomes? and (3) What study designs and measurement techniques have been employed to investigate the effects of footwear in trail running and hiking research?

## 2 Methods

A scoping review methodology was selected to map the breadth and characteristics of evidence on trail running and hiking footwear, where heterogeneity in populations, interventions, and outcomes precluded a meaningful synthesis of findings. The review was conducted using the framework developed by Arksey et al. [19], and reported in accordance with the Preferred Reporting Items for Systematic Review and Meta‐Analyses extension for Scoping Reviews (PRISMA‐ScR) guidelines [20]. The review question, eligibility criteria, and data charting approach were developed a priori, informed by JBI guidance for scoping reviews. No formal protocol was registered; however, the review methods were established a priori. The search strategy was developed collaboratively by all authors and conducted across four databases: Scopus, Medline (via EBSCO), SPORTDiscuss, and CINAHL. Searches were performed on 15th May 2025, with no restriction on publication date. An initial search strategy was developed for Scopus (Table 1) and iteratively refined. This strategy was then adapted for each database to account for differences in database syntax and functionality (e.g., truncation symbols). No controlled vocabulary or subject headings were used. The full search strategies for all databases, including search strings, interfaces, and limits, are provided in Supporting Information S1.

**TABLE 1:** Example search strategy (used for Scopus^a^).

#	Search
1	TITLE‐ABS‐KEY (footwear OR shoe* OR boot)
2	TITLE‐ABS‐KEY (trail OR mountain OR off‐road OR fell)
3	TITLE‐ABS‐KEY (run* OR hik* OR walk*)
4	#2 AND #3
5	#1 AND #4

^a^ Truncation was adapted for each database—full details of search available in Supporting Information S1

Eligibility criteria were defined a priori in line with a Population–Concept–Context (PCC) framework. The population included adult participants (≥ 18 years) engaged in trail running or hiking. The concept of interest was the influence of footwear, including shoes or boots, on relevant outcomes. The context was restricted to trail running and hiking activities. Studies were included if they reported data relating to the influence of footwear on biomechanical, physiological, performance, comfort, or injury outcomes. Studies were excluded if they focused on activities other than hiking and trail running, did not analyse footwear, or were not published in English.

Duplicate records were initially removed automatically using EndNote (v21.0.1, Clarivate Analytics, PA, USA), followed by manual verification. Two reviewers (AJ and KH) independently screened all titles and abstracts for relevance. Disagreements at each stage were resolved through discussion, with a third reviewer available if consensus could not be reached; however, this was not required. Subsequently, the same reviewers assessed the full texts of the remaining articles to determine final eligibility. Data charting was undertaken using a standardised extraction form developed by the author team in Microsoft Excel (Microsoft Corp., WA, USA). The form captured key study characteristics, including study design, participant characteristics, activity type (hiking or trail running), footwear type, outcome measures, and key findings. The data charting form was initially piloted on a small sample of included studies and refined iteratively to ensure consistency and completeness. Data extraction was conducted independently by two reviewers (AJ and KH), with discrepancies resolved through discussion.

Data were synthesised descriptively to map the distribution and characteristics of the evidence. Findings were summarised using tabulation and grouped according to key outcome domains. No formal critical appraisal was undertaken.

## 3 Results

### 3.1 Selection and Characteristics of Studies

**FIGURE 1:**
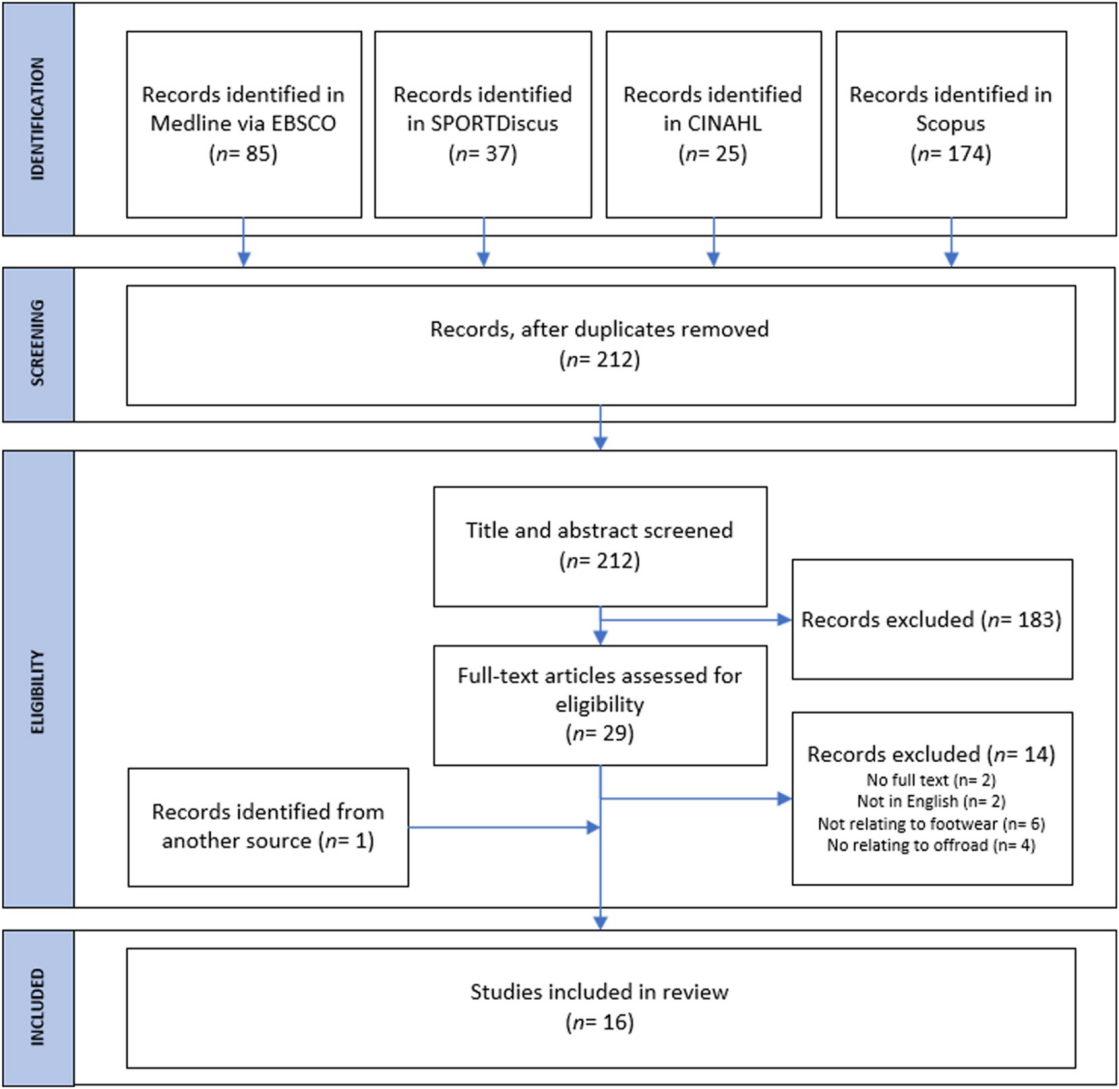
Flowchart of the literature search and screening process.

Following the removal of duplicates, 212 studies were screened, of which 15 met the inclusion criteria for final analysis (Figure 1). Prior to submission, a final check of recently published literature was undertaken to identify any additional eligible studies. This identified one additional study [21], which was assessed against the predefined eligibility criteria and included, bringing the total number of studies to 16. Thirteen studies (81%) focused on trail running [4, 6, 15, 21, 22, 23, 24, 25, 26, 27, 28, 29, 30] whilst the remaining 3 studies (19%) related to hiking [5, 11, 12]. Studies were published between 2009 and 2025 and employed diverse methodologies, including cross‐sectional surveys [5, 6, 11], crossover trials [4, 15, 21, 27, 28, 30], repeated‐measures designs [22, 23, 24, 25], and observational studies [12, 26, 29]. The focus of each study (injury, biomechanics, physiological, performance, comfort) is outlined in Table 2.

**TABLE 2:** Outcome measures from the included studies.

Author (year)	Hike/run	Outcome measures				
		Injury	Biomechanical	Physiological	Performance	Comfort
Anderson et al. [5]	Hike	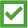	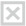	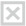	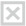	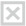
Chicharro‐Luna et al. [12]	Hike	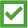	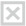	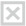	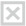	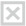
Corbí‐Santamaría et al. [30]	Run	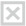	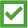	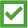	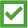	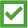
Fukuchi et al. [4]	Run	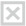	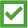	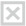	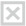	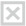
Hamill et al. [6]	Run	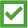	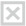	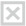	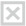	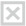
Hintzy et al. [29]	Run	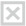	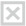	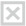	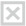	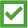
Honert et al. [28]	Run	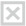	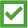	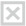	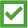	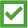
Inomata [27]	Run	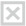	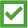	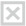	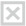	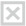
Jaboulay and Giandolini [15]	Run	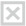	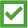	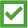	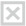	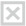
Kasmer et al. [26]	Run	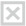	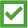	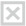	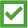	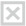
Lloria‐Varella et al. [25]	Run	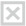	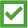	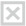	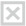	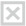
Mo et al. [24]	Run	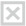	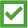	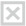	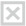	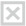
Muzeau et al. [21]	Run	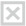	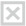	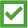	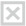	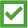
Soraruf et al. [23]	Run	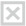	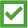	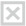	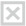	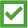
Uno et al. [11]	Hike	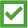	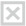	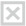	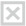	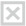
Vercruyssen et al. [22]	Run	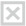	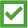	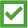	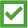	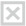

Participant numbers ranged from 2 to 1061 (mean 177 ± 336) with a distinct difference between survey/observational research (mean 536 ± 415 participants), and experimental methodologies (mean 13 ± 7). The mean age of participants ranged from 21 to 39 years. Male participants were more common than female, with six studies (38%) including only males [4, 15, 21, 22, 27, 30], a further three studies (19%) including mostly (66%–71%) male participants [5, 11, 26], and only one study [6] including more females than males (52% female).

The 16 studies analysed various footwear considerations and characteristics. These included shoe type (e.g., hiking boots, trail shoes, non‐trail shoes) [5, 11, 12], AFT footwear [4, 15, 21, 30], minimalist versus conventional designs [22, 24, 26], midsole characteristics [21, 23, 25], tread patterns [27], and closure systems (lacing or wrap styles) [28].

### 3.2 Biomechanics

Ten studies (62%) included biomechanical outcomes [4, 15, 22, 23, 24, 25, 26, 27, 28, 30], which included kinetic [4, 15, 25, 27, 28], spatiotemporal [25, 30], plantar pressure [4], vertical stiffness [22, 25, 30], strike position [22, 24, 26], kinematic [15, 27], and tibial acceleration [23, 24]. The key biomechanical findings of these studies are outlined in Table 3.

**TABLE 3:** Key methods and findings of studies with biomechanical outcomes.

Author (year)	Cohort description	Participant number (% male)	Mean participant age (SD)	Footwear type/comparison	Setting	Biomechanical assessment method	Key findings
Corbí‐Santamaría et al. [30]	Mountain runners—well trained with at least 3 years' experience. Average weekly mileage 61 km	12 (100)	35.4 (9.5)	Comparison between conventional mountain running shoes and advanced footwear technology shoes (AFT). footwear was matched for mass but AFT had higher heel and forefoot height.	Simulated mountain race on an outdoor trail circuit (5.19 km, uphill, downhill and mixed sections) for time trials.	Vertical stiffness (pre/post trail run). Biomechanical variables recorded during running: Step frequency (spm), step length (cm), contact time (ms), leg stiffness (kN/m), and vertical oscillation of the centre of gravity (cm).	Footwear significantly reduced step frequency (*P* = 0.04) and increased vertical oscillation (*P* = 0.01) during the uphill segment.
Fukuchi et al. [4]	Runners familiar with trail running	11 (100)	29.0 (5.6)	Control (no carbon plate insole) versus Carbon plate (insole inserted).	Outdoor trail running. Tested uphill (18.5% incline) and downhill (17.2% incline).	Wearable IMU sensors (on shank and foot) measured peak axial acceleration. In‐shoe plantar pressure sensor measured peak maximum pressure across four regions (toe, metatarsal, midfoot, heel).	Carbon fibre plate resulted in a decreased peak shank axial acceleration during uphill running compared to control (*P* = 0.008). No significant difference in shank acceleration downhill (*P* = 0.066). Peak plantar pressure was not influenced by shoe condition, regardless of slope.
Honert et al. [28]	Trail runners with an average weekly mileage of 24 km.	29 (52)	34 (8)	Comparison between wrap closure (La Sportiva Cyklon) and a lace closure (retrofitted La Sportiva Cyklon)	Outdoor trail loop (1.6 km), segmented into uphill, top technical, and downhill sections.	IMU sensors measured peak eversion angular velocity, peak acceleration, jerk, and medial‐lateral acceleration ranges. Plantar pressure insoles measured average heel and toe contact area, and peak heel/toe pressures.	Peak eversion velocity was 5% lower in the wrap shoe for uphill (*P* = 0.025), top technical (*P* = 0.008), and downhill (*P* = 0.043). Average heel contact area increased by 2% in the wrap shoe for all sections (*P* < 0.001). Other acceleration and pressure metrics showed no significant differences.
Inomata [27]	Trail runners	2 (100)	34 (12.7)	Different outer sole tread arrangements (e.g., Type‐1, Type‐2) tested on a prototype shoe.	Laboratory setting simulating running on a hard clay (muddy surface condition).	3D motion capture system measured shoe slip behaviours and maximum slip distance. Force plate measured horizontal GRF (Fy). Analysis of tread position relative to the centre of pressure.	Shoe grip is enhanced by designing the forefoot area based on both tread position (corresponding to CoP at 60% stance—the beginning of the slip at propulsion) and tread numbers.
Jaboulay and Giandolini [15]	Amateur but experienced trail runners	10 (100)	32 (8)	Control prototype (LBS: 11.0 N/mm) versus Plated prototype (LBS: 16.5 N/mm).	Laboratory setting on a 20‐m uneven track (simulating unstable terrain).	3D motion capture and force plates measured 3D kinematics and kinetics (ranges of motion, moment peaks). Calculated surrogates for joint control: Normalised Jerk (nJ).	No footwear effect was observed for any biomechanical discrete variables (e.g., contact time *P* = 0.154; sagittal foot strike angle *P* = 0.913).
Kasmer et al. [26]	Trail runners participating in a 50 km trail race.	161 (71)	NR	Comparison between minimalist shoe type (offset 4 mm or less) and traditional shoe type.	In‐race video recording at the 8.1 km mark of a 50 km trail race.	Video analysis (240 fps) allowed foot strike classification (rear‐foot strike, mid‐foot strike, fore‐foot strike).	RFS prevalence was 85.1%. A significant effect of shoe type on foot strike was observed: RFS was less common among runners in minimalist shoes (*P* < 0.01).
Lloria‐Varella et al. [25]	Runners who completed a 38 km trail race.	18 (50)	37 (8)	Personal shoes (worn/fatigued) versus control shoes (new/non‐used).	Laboratory treadmill (with belt stiffeners) pre‐ and post‐race. Race conducted on a natural trail.	OptoJump measured spatiotemporal parameters (e.g., contact time, step frequency). Triaxial accelerometer measured tibial peak‐to‐peak amplitude (R, AP, Vert, ML axes). Spring‐mass parameters vertical stiffness and leg stiffness were calculated.	Footwear fatigue did not interact with spatiotemporal parameters or leg stiffness. A significant interaction was found for peak‐to‐peak amplitude ML (*P* < 0.05)
Mo et al. [24]	Trail runners who habitually rearfoot strike	18 (50)	38.5 (9.6)	Minimalist (Newton MV2) versus maximalist (Hoka Clifton 3)	In‐field running on a natural trail (level, uphill, and downhill slopes).	Accelerometers measured peak tibial acceleration. Pressure sensing insoles measured strike index (location of COP at initial contact).	No footwear effect (minimalist vs. maximalist) on peak tibial acceleration (*pP* = 0.27) or strike index (*pP* = 0.056).
Soraruf et al. [23]	Experienced trail runners	7 (NR)	29 (7.6)	Eight commercially available trail‐running shoes covering a range of nominal cushioning levels.	Combination of indoor treadmill running (at 3 cadences: 155, 175, 195 steps/min) and outdoor overground running on soft and gravel road (level and downhill).	Inertial measurement units fixed on the tibia and foot measured linear acceleration. Key metrics extracted: Median frequency and median frequency during the first 25% of the stance, average power of spectrum, and acceleration peaks.	The median frequency of the tibial acceleration during the first 25% of the stance was the most relevant metric, which decreased with increasing cushioning during indoor running. Impact peak force measured in the lab was inversely associated with perceived cushioning.
Vercruyssen et al. [22]	Competitive trail runners with limited experience in minimal footwear. Mean VO2 max 62.5	13 (100)	38.2 (4.8)	Comparison among traditional shoes (Salomon XT Wings, 10 mm offset), minimalist shoes (Salomon Sense, 4 mm offset), and minimalist shoes plus added mass (to match TS mass).	Combined setting: Outdoor 18 km trail course to induce neuromuscular fatigue, and laboratory motorised treadmill for pre‐ and post‐ trail run physiological tests.	High‐speed camera allowed for measurement of foot strike angle. Vertical stiffness (the ratio of max ground reaction force to max downward displacement of centre of mass) and leg stiffness (ratio of max ground reaction force to peak displacement of leg spring) were calculated.	Foot strike angle was significantly lower (more mid‐foot strike/flatter foot) in minimalist and minimalist plus mass compared with traditional shoes during level running (both pre and post trail run) (*P* = 0.004). Vertical stiffness (*P* = 0.030) and leg stiffness (*P* = 0.031) were significantly lower in minimalist and minimalist plus mass compared with traditional shoes.

Biomechanical outcomes were gathered both in a laboratory setting [15, 22, 23, 25, 27] and outside on trails [4, 23, 24, 26, 28, 30]. Spatiotemporal parameters were frequently gathered using optical systems (OptoJump) or inertial measurement units (IMUs) fixed to the shank or rearfoot. These outcomes included the measurement of step frequency (steps/min), step length (cm), contact time (ms), and aerial time. Corbí‐Santamaría et al. [30] specifically monitored the vertical oscillation of the body centre of gravity (cm). Kinematic analysis was conducted using marker‐based three‐dimensional (3D) motion capture to track segment trajectories (feet, shanks, thighs, and pelvis). The resulting kinematics included discrete variables such as joint ranges of motion and peak angles.

Kinetics and vertical/leg stiffness were typically assessed using force plates, which recorded 3D ground reaction forces (GRF) sampled at high frequencies (e.g., 2000 Hz). Metrics derived from these data included peak moments, sagittal power outputs and inputs, and horizontal GRF when analysing slip on simulated surfaces (such as wet clay on a board within a lab). Vertical and leg stiffness were calculated using the spring‐mass model, requiring contact and aerial time data. Impact and shock attenuation were primarily quantified using IMUs. The primary metric for impact loading was peak tibial acceleration, sometimes reported as peak axial acceleration (on the shank and foot). Acceleration signals were analysed in both the time and frequency domains. Time‐domain metrics included peak acceleration (shock) and peak jerk magnitude. Frequency‐domain analysis involved extracting the median frequency and median frequency during the first 25% of the stance, metrics suggested to correlate with tissue resonance and cushioning. Joint control and smoothness were assessed using the third derivative of displacement, quantified as the Normalised Jerk (nJ) derived from knee and ankle frontal and transverse movements.

Foot strike angle or pattern was defined and classified in five studies [4, 22, 24, 26, 30] using different methods. Foot strike angle was determined visually from high‐speed video recordings, measuring the angle between the treadmill belt and a line connecting the fifth metatarsal joint and the calcaneus [22]. Strike index was used in other studies to define the foot strike pattern based on the location of the centre of pressure (COP) at initial contact along the long axis of the foot. In‐shoe plantar pressure sensors (XSENSOR, Pedar‐X) were used to measure peak maximum pressure across specific foot regions (toe, metatarsal, midfoot, heel), as well as the average heel and toe contact area. Footwear effectiveness in unstable terrain was measured by Inomata [27] via shoe slip behaviour, quantifying maximum slip distance using 3D motion capture.

### 3.3 Comfort

Table 4 presents outcome measures and key findings from the five studies (31%) which investigated the impact of footwear on comfort during trail running [21, 23, 28, 29, 30]. Primarily, these studies have used subjective scales to measure overall feeling, fit and comfort. Overall comfort and fit were measured using a visual analogue scale (VAS), ranging from 100 to 150 mm. Participants also rated specific fit characteristics and individual comfort dimensions via VAS, such as heel cushioning, forefoot cushioning, shoe stability, forefoot flexibility, and shoe grip.

**TABLE 4:** Key methods and findings of studies with comfort outcomes.

Author (year)	Cohort/setting description	Participant number (% male)	Mean participant age (SD)	Footwear type/comparison	Setting	Comfort assessment method	Key findings
Corbí‐Santamaría et al. [30]	Mountain runners—well trained with at least 3 years' experience. Average weekly mileage 61 km	12 (100)	35.4 (9.5)	Comparison between conventional mountain running shoes and advanced footwear technology shoes (AFT). Footwear was matched for mass but AFT had higher heel and forefoot height.	Simulated mountain race on an outdoor trail circuit (5.19 km, uphill, downhill and mixed sections) for time trials.	Overall comfort and specific characteristics (heel cushioning, forefoot cushioning, stability, flexibility, grip) rated using a 100‐mm visual analogue scale.	Overall comfort was unchanged between shoes. Footwear significantly affected the subjective perception of flexibility. AFT shoes were perceived as less flexible than conventional shoes.
Hintzy et al. [29]	Trail runners	10 (NR)	21.1 (1)	Same model of new trail running footwear (Salomon XT Wings) used for all subjects.	Outdoor trail course (13 km, 5 laps of 2.6 km) consisting of flat, uphill, and downhill sections.	Overall footwear comfort evaluated using a 150‐mm visual analogic scale before running (lap 0) and at the end of each lap (lap 1–5).	Overall footwear comfort decreased consistently during the run session (*P* < 0.001). The decrease in overall footwear comfort became significant only after 44 min of running (7.8 km). The total overall footwear comfort decrease was substantial (32%).
Honert et al. [28]	Trail runners with an average weekly mileage of 24 km.	29 (52)	34 (8)	Comparison between wrap closure (La Sportiva Cyklon) and a lace closure (retrofitted La Sportiva Cyklon)	Outdoor trail loop (1.6 km), segmented into uphill, top technical, and downhill sections.	Ordinal scales (0–10) for overall fit, specific forefoot/midfoot/heel fit (where 0 = ‘poor’, and 10 = ‘great’).	The wrap shoe was rated better for: overall fit (*P* < 0.001). Wrap was rated as tighter in the forefoot (*P* = 0.04).
Muzeau et al. [21]	Well trained athletes accustomed to running uphill and downhill. Average weekly mileage 79 km	14 (100)	29.4 (7.3)	Comparison between a shoe with traditional foam (stiffer) and a shoe with AFT foam (more compliant and resilient). Both models had identical geometry and included a plate.	Laboratory setting using a treadmill for all assessments across flat (0% incline, 14 km.h^−‐1^), uphill (+10% incline, 8 km.h^−‐1^), and downhill (−10% incline, 14 km.h^−‐1^) gradients.	Perceived effort (Borg 6–20 scale). Affective valence (pleasure/displeasure, feeling scale −5 to 5). Arousal (activation, felt arousal scale 0–6).	Rate of perceived exertion was lower in AFT foam compared to traditional foam (*P* = 0.008). Affective valence was more positive (increased pleasure) in AFT foam versus traditional foam (*P* = 0.027). Arousal showed no difference between shoes (*P* = 0.738$).
Soraruf et al. [23]	Experienced trail runners	7 (NR)	29 (7.6)	Eight commercially available trail‐running shoes covering a range of nominal cushioning levels.	Combination of indoor treadmill running and outdoor overground running on soft and gravel road (level and downhill).	Perceived cushioning rated by a sensory‐trained panel on a 0–100 visual analogue scale (0 min, 100 max cushioning).	Trained subjects could discriminate different levels of cushioning (*P* < 0.001). Perceived cushioning correlated strongly with biomechanical outcomes (e.g., lower median frequency of tibial acceleration correlated with higher perceived cushioning). Cushioning differences were reported to be better perceived during downhill running compared to level running.

### 3.4 Physiology

Physiological assessments conducted by four studies (25%) [15, 21, 22, 30] primarily focused on quantifying metabolic efficiency and markers of neuromuscular fatigue. The key outcome measures and findings for these studies are presented in Table 5. Running economy (RE) was measured as oxygen consumption and carbon dioxide production collected continuously using a portable metabolic cart. Metabolic power (W/kg) was calculated, normalising energy expenditure by body mass. Cardiovascular and exertion heart rate (beats/min) was collected continuously using chest sensors (e.g., Polar H10) or optical heart rate sensors. Heart rate was used as both a physiological metric related to RE and an objective measure of exertion to ensure consistency of effort across conditions. Biochemical fatigue markers included blood lactate concentrations (mMol/L), measured from the fingertips pre‐ and post‐exercise. One study objectively confirmed neuromuscular fatigue induced by prolonged running by measuring the reduction in maximal voluntary contraction torque of the knee extensors using an isometric dynamometer [22].

**TABLE 5:** Key methods and findings of studies with physiological outcomes.

Author (year)	Cohort description	Participant number (% male)	Mean participant age (SD)	Footwear type/comparison	Setting	Physiological assessment method	Key findings
Corbí‐Santamaría et al. [30]	Mountain runners—well trained with at least 3 years' experience. Average weekly mileage 61 km	12 (100)	35.4 (9.5)	Comparison between conventional mountain running shoes and advanced footwear technology shoes (AFT). Footwear was matched for mass but AFT had higher heel and forefoot height.	Simulated mountain race on an outdoor trail circuit (5.19 km, uphill, downhill and mixed sections) for time trials.	Pre/post‐test blood lactate (fingertips); continuous heart rate (chest strap)	The use of AFT did not improve physiological responses during the simulated mountain race.
Jaboulay and Giandolini [15]	Amateur runners who ran trails at least once per week	10 (100)	32 (8)	Comparison between a control prototype (low longitudinal bending stiffness, LBS: 11.0 N/mm) and a plated prototype (increased LBS: 16.5 N/mm). Shoes were matched for stack height and drop, but not mass.	Laboratory setting, treadmill. Tests were conducted at level (1% incline) and uphill (10% incline) gradients.	Metabolic energy expenditure (metabolic power, W/kg) calculated from average oxygen consumption and carbon dioxide production, collected via a portable gas exchange analyser during 5‐min bouts on a treadmill.	No footwear effect on metabolic power was observed during level running. A significant 2% increase (degradation) in metabolic energy expenditure was observed in the plated condition when running uphill (*P* = 0.04).
Muzeau et al. [21]	Well trained athletes accustomed to running uphill and downhill. Average weekly mileage 79 km	14 (100)	29.4 (7.3)	Comparison between a shoe with traditional foam (stiffer) and a shoe with AFT foam (more compliant and resilient). Both models had identical geometry and included a plate.	Laboratory setting using a treadmill for all assessments across flat (0% incline, 14 km h^−‐1^), uphill (+10% incline, 8 km h^−‐1^), and downhill (−10% incline, 14 km h^−‐1^) gradients.	Running economy collected continuously via a portable metabolic cart during 6‐min treadmill stages at three gradients. Heart rate collected via a chest sensor.	Oxygen consumption was 1.2% lower in the shoe with AFT foam compared to that with traditional foam (*P* = 0.008) regardless of gradient, indicating improved running economy. Heart rate was lower in AFT foam compared to traditional foam (*P* < 0.001).
Vercruyssen et al. [22]	Competitive trail runners with limited experience in minimal footwear. Mean VO2 max 62.5	13 (100)	38.2 (4.8)	Comparison among traditional shoes (Salomon XT Wings, 10 mm offset), minimalist shoes (Salomon Sense, 4 mm offset), and minimalist shoes plus added mass (to match TS mass).	Combined setting: Outdoor 18 km trail course to induce neuromuscular fatigue, and laboratory motorised treadmill for pre‐ and post‐ trail run physiological tests.	Running economy, expressed as gross energy cost (J kg^−‐1^.m^−‐1^), calculated from breath‐by‐breath oxygen consumption (VO_2_) values during 5‐min treadmill stages (level and uphill) pre‐ and post‐trail run. Heart rate monitored continuously during the 18.4 km course.	In the non‐fatigued state (pre‐trail run), runners exhibited better RE during level running in the minimalist and minimalist plus mass shoes compared with traditional shoes (*P* = 0.032). In the fatigued state (post trail run), RE was altered (impaired) during level running in both minimalist and minimalist plus mass. No significant difference in RE was observed between shod conditions during uphill running.

### 3.5 Performance

Performance outcomes were explored in four studies (25%) [22, 26, 28, 30]. Time, speed, and pacing performance were assessed by recording the total time to complete specific trail courses. Running speed (m/s or km/h) was controlled during laboratory treadmill testing or measured continuously via IMU/GPS during field tests. Kasmer et al. [26] assessed runner performance based on their rank (defined as their placement relative to other competitors) at the 8 km checkpoint during a 50 km trail race. Corbí‐Santamaría et al. [30] obtained mean power output and maximum power output using an IMU based power metre (Stryd). Performance outcome measures and relevant results of the included studies are outlined in Table 6.

**TABLE 6:** Key methods and findings of studies with performance outcomes.

Author (year)	Cohort description	Participant number (% male)	Mean participant age (SD)	Footwear type/comparison	Setting	Performance assessment method	Key findings
Corbí‐Santamaría et al. [30]	Mountain runners—well trained with at least 3 years' experience. Average weekly mileage 61 km	12 (100)	35.4 (9.5)	Comparison between conventional mountain running shoes and advanced footwear technology shoes (AFT). Footwear was matched for mass but AFT had higher heel and forefoot height.	Simulated mountain race on an outdoor trail circuit (5.19 km, uphill, downhill and mixed sections) for time trials.	Total time (*ss*) for the circuit; segment times (*ss*) (uphill, downhill, mixed); mean/maximum power (W) (Stryd power metre); Fastest km (s/km).	AFT did not influence overall performance or time. In the downhill segment, the fastest kilometre performance was slower in the AFT shoe.
Honert et al. [28]	Trail runners with an average weekly mileage 24 km.	29 (52)	34 (8)	Comparison between wrap closure (La Sportiva Cyklon) and a lace closure (retrofitted La Sportiva Cyklon)	Outdoor trail loop (1.6 km), segmented into uphill, top technical, and downhill sections.	Running speed (m/s) determined via IMU sensors for the whole loop and for each section.	Runners ran slightly but significantly faster in the wrap shoe (average difference: 0.03–0.05 m/s, *P* < 0.022) on all three trail sections.
Kasmer et al. [26]	Trail runners participating in a 50 km trail race.	161 (71)	NR	Comparison between minimalist shoe type (offset 4 mm or less) and traditional shoe type.	In‐race video recording at the 8.1 km mark of a 50 km trail race.	Race performance assessed by rank at 8.1 km. Finishing times (pace range) were also noted for context.	A significant effect of shoe type on performance was observed: Faster runners were more likely to be wearing minimalist shoes (*P* < 0.01).
Vercruyssen et al. [22]	Competitive trail runners with limited experience in minimal footwear. Mean VO2 max 62.5	13 (100)	38.2 (4.8)	Comparison among traditional shoes (Salomon XT Wings, 10 mm offset), minimalist shoes (Salomon Sense, 4 mm offset), and minimalist shoes plus added mass (to match TS mass).	Combined setting: Outdoor 18 km trail course to induce neuromuscular fatigue, and laboratory motorised treadmill for pre‐ and post‐ trail run physiological tests.	Running time (*s*) over the 18.4 km trail course monitored via GPS watches.	No significant variation in running time was observed between the three footwear conditions over the trail course.

### 3.6 Injury

Table 7 presents an overview of the means used to assess injury in trail running and hiking as well as the key findings. Injury assessment relied on retrospective surveys for prevalence [5, 6, 11] and clinical examination for acute dermal lesions (blisters) [12]. Injury outcomes were predominantly investigated via retrospective self‐reported questionnaires completed by runners or hikers. Survey questions used yes/no or multiple‐choice formats. Injuries spanned both traumatic and chronic conditions, including the prevalence of specific outcomes, such as a rolled or sprained ankle (the most reported traumatic injury), knee pain, shin pain, and plantar fasciitis.

**TABLE 7:** Key methods and findings of studies with injury outcomes.

Author (year)	Cohort/setting description	Participant number (% male)	Mean participant age (SD)	Footwear type/comparison	Injury assessment method	Key findings
Anderson et al. [5]	Long distance hikers who walked the appalachian or pacific Crest trails	128 (70)	32.7 (11.4)	Comparison of footwear based on increasing rigidity: Sandals, running shoes, low‐top hiking shoes, and hiking boots.	Cross‐sectional self‐reported survey administered at designated stations. The primary outcome analysed was the prevalence of paraesthesia. Other injuries and illnesses were also collected via yes/no or multichoice questions.	In univariate analysis, increasing footwear rigidity was associated with increasing prevalence of paraesthesia (29% for sandals vs. 68% for hiking boots). However, after adjusting for pack weight in multivariate analysis, footwear type did not have a statistically significant effect on the prevalence of paraesthesia (*P* = 0.16). Footwear was not associated with other musculoskeletal injuries.
Chicharro‐Luna et al. [12]	Long distance hikers receiving podiatry attention along the Way of St. James in Spain	315 (53)	36.0 (14.1)	Footwear was categorised as hiking boot, hiking shoe, hiking sandal, sports shoe, or trail‐running shoe. Comparison also included new versus used footwear, footwear weight, and the use of foot orthoses.	Cross‐sectional observational study involving clinical interviews and clinical examination by podiatrists. The primary injury outcome was the presence and location of blisters on the foot.	74% of hikers presented a blister. Footwear type was not associated with the development of blisters. The use of foot orthoses was identified as a protective factor against blistering (*P* = 0.001). Distance walked on asphalt and having wet socks at the end of the day were identified as risk factors.
Hamill et al. [6]	Runners in the United States who run on trails at least once per month	1016 (48)	39.0 (13.2)	Comparison between wearing footwear specifically designed for trail running versus other shoes (e.g., road running shoes). The perceived importance of various shoe characteristics (e.g., traction, cushion, protection) was also assessed.	Online retrospective self‐reported questionnaire. Runners reported all injuries they had sustained while trail running. Injury types included traumatic injuries (e.g., fractures, ankle sprain) and chronic injuries (e.g., back pain, plantar fasciitis).	The most reported injury was a sprained ankle. In total, 39.8% of respondents reported a trail running injury occurrence. No significant association was found between injury occurrence and the type of footwear worn (trail‐specific vs. other). Significant associations were found between injury occurrences and running on highly technical, exposed, sandy, or muddy terrain.
Uno et al. [11]	Hikers who had descended Mt Fuji, Japan	1061 (66)	37.0 (14.0)	Comparison between hiking shoes or mountaineering boots versus other types of shoes (e.g., running shoes, sneakers). The study also assessed the subjective condition of the shoe sole (not worn out, moderately worn out, quite worn out).	Questionnaire survey (self‐reported) of hikers. Falls were defined as ‘ground contact with any portion of the body other than the feet’. The survey documented the incidence of falls, motion of falls, and injury status when falling (e.g., none, knee pain, sprained ankle, scratch).	The fall rate was higher in women (49%) than in men (35%). Factors that decreased the risk of falls for the overall group included: Wearing hiking shoes or mountaineering boots rather than other types of shoes (OR 0.812) and having shoe soles that were not worn out (OR 1.252 suggests worn‐out increases risk).

Several specific symptoms or events were investigated, including paraesthesia, defined as ‘burning, tingling or numbness in the arms, legs or feet’, which was investigated in relation to shoe type and pack weight by Anderson et al. [5]. Chicharro‐Luna et al. [12] used an interview and clinical examination by qualified podiatrists to record the presence and specific location (e.g., toes, metatarsal heads, heel) of blisters. Falls in mountaineering were studied by Uno et al. [11] examining hikers who had descended Mt Fuji. A fall was explicitly defined as ‘ground contact with any portion of the body other than the feet’. Information related to the number of falls, the motion of falls (stagger, stumbling, slip, caught pole, and others), and the immediate injury status when they fell (none, knee pain, sprained ankle, scratch, and others) was collected.

## 4 Discussion

The current body of research on footwear in trail running and hiking demonstrates a commendable effort to evaluate the complex human‐footwear interaction across varied environments, yielding significant methodological strengths, while simultaneously highlighting persistent limitations and clear evidence gaps. A key strength is the commitment to ecological validity in trail running, with many studies employing blended protocols that combine controlled laboratory assessments (e.g., metabolic testing, mechanical stiffness) with multi‐kilometre runs on natural trails. This approach, particularly by leveraging wearable sensors, provides insights into running biomechanics specific to the ecologically demanding nature of trails that cannot be captured in highly controlled lab environments. For instance, Corbí‐Santamaría et al. [30] used a simulated mountain running event, while Fukuchi et al. [4] and Honert et al. [28] conducted in‐field testing with IMUs and pressure sensors, offering a higher level of ecological validity compared to studies focused solely on treadmills. Similarly, the epidemiological research involving large‐scale cross‐sectional surveys of trail runners [6] and long‐distance hikers [5] contributes valuable, albeit self‐reported, data on injury prevalence and risk factors across large populations. This push for real‐world data is offset by the pervasive finding that most IMU‐based studies remain highly controlled; Benson et al. [31] found that the majority (72%) of these studies were conducted indoors, typically on a treadmill at prescribed speeds, and over small distances. This reality contrasts sharply with the recognised capability of IMUs to capture running biomechanics in less controlled settings.

A major limitation across the research is the frequent use of small sample sizes (often *N* < 15), which restricts the statistical power needed to confidently detect subtle or complex effects and limits the generalisability of findings, particularly to female populations, who are underrepresented in many controlled trials [32]. Furthermore, achieving high levels of experimental control is challenging; for instance, while some studies demonstrated methodological rigour by comparing shoes from the same manufacturer that varied only in key characteristics [15, 21], many other comparison studies use shoes from different brands, introducing multiple uncontrolled confounding factors. The reliance on commercial sensors also introduces variability; comparative studies have shown that different popular wearable devices (e.g., Stryd vs. GARMIN) exhibit systematic bias and poor agreement for key metrics such as power and vertical oscillation, highlighting the current absence of a universal gold standard for real‐world trail conditions [33].

One of the most significant evidence gaps concerns understanding prolonged footwear use and its interaction with fatigue. Most studies focus on acute effects, ignoring that fatigue alters kinematics, kinetics, and energetics [34]. Even research incorporating race‐induced fatigue protocols suffers from limitations, such as using runners' personal shoes with uncontrolled prior mileage [25], which confounds the effects of runner fatigue with potential shoe material deterioration [22]. Similarly, the laboratory simulation of technical running, particularly the downhill condition, proved difficult, inducing high participant discomfort that potentially interfered with obtaining representative physiological loads [21]. The reliance on retrospective self‐reported data in injury studies [6] also introduces limitations, lacking medical or biomechanical confirmation for the injury type or aetiology.

Given the limitations and gaps identified in the current evidence base, future research may benefit from progressing towards evidence‐based individualisation of footwear, recognising that individual responses are highly variable [35]. This would likely require further methodological development, as proposed by Willwacher et al. [36], including the establishment of standardised protocols for measurement (e.g., marker sets, joint definitions, filtering processes) to enable the collection of large datasets and the application of machine learning analysis. Future models may seek to integrate individual, task, and environmental variables to predict the optimal footwear choice. Potential methodological innovations may include leveraging 3D printing to systematically vary experimental footwear components in controlled trials. Finally, future studies may benefit from moving beyond acute trials towards longitudinal designs to better understand the chronic effects of trail running and hiking footwear on the lower limb.

## 5 Conclusion

This scoping review systematically mapped the complex interaction between humans and footwear in dynamic off‐road environments, finding that existing research strongly prioritises acute biomechanical outcomes in trail running, often leveraging wearable sensors in blended laboratory and field protocols to enhance ecological validity. A key methodological limitation remains the persistent use of small sample sizes in experimental trials and the underrepresentation of female participants, both of which restrict the generalisability of findings. Furthermore, a significant knowledge gap exists regarding the chronic effects of prolonged footwear use and how footwear characteristics interact with fatigue. To mature this field, future research requires methodological standardisation, including protocols for sensor use and measurement techniques, to support the collection of larger data sets and machine learning applications that advance toward individualised, evidence‐based footwear solutions. Ultimately, advancing the understanding of off‐road footwear requires a multidisciplinary effort, incorporating materials science, and set alongside longitudinal studies that move beyond acute measures.

## Author Contributions

**Aaron Jackson:** conceptualization, formal analysis, data curation, methodology, writing – original draft, writing – review and editing. **Kiseon Hong:** conceptualization, data curation, methodology, writing – review and editing. **Mike Frecklington:** conceptualization, methodology, writing – review and editing. **Kelly Sheerin:** conceptualization, methodology, writing – review and editing. **Matthew Carroll:** conceptualization, methodology, writing – review and editing.

## Funding

The authors have nothing to report.

## Conflicts of Interest

The authors declare no conflicts of interest.

## Supporting information


Supporting Information S1

## Data Availability

No new data were generated or analysed for this study. This article is a scoping review based on previously published literature.
