# Supplementary material for: Exploring the Role of Footwear in Trail Running and Hiking: A Scoping Review
Source: J Foot Ankle Res. 2026 Aug 8;19(3):e70197. doi: 10.1002/jfa2.70197 (PMC13452467; doi:10.1002/jfa2.70197)
Supplement: Supplementary file 1 [file JFA2-19-e70197-s001.docx]

# Supplementary Information 1: Full Search Strategies

## Overview

The search strategy was developed collaboratively by the author team and designed to identify studies investigating the influence of footwear in trail running and hiking. An initial strategy was developed for Scopus and subsequently adapted for use across other databases, accounting for differences in syntax and functionality. Searches were conducted on 15 May 2025 across all databases. No limits were applied for publication date. Only studies published in English were considered at the screening stage. No controlled vocabulary (e.g., MeSH headings) was used; all searches were conducted using keyword-based strategies.

## Database: Scopus (Elsevier)

Date searched: 15 May 2025

Limits applied: None

*(footwear OR shoe* OR boot)*

*AND*

*(trail OR mountain OR off-road OR fell)*

*AND*

*(run* OR hik* OR walk*)*

## Database: MEDLINE (EBSCO)

Date searched: 15 May 2025

Limits applied: None

*(footwear OR shoe* OR boot*)*

*AND*

*(trail OR mountain OR off-road OR fell)*

*AND*

*(run* OR hik* OR walk*)*

## Database: SPORTDiscus (EBSCO)

Date searched: 15 May 2025

Limits applied: None

*(footwear OR shoe* OR boot*)*

*AND*

*(trail OR mountain OR off-road OR fell)*

*AND*

*(run* OR hik* OR walk*)*

## Database: CINAHL (EBSCO)

Date searched: 15 May 2025

Limits applied: None

*(footwear OR shoe* OR boot*)*

*AND*

*(trail OR mountain OR off-road OR fell)*

*AND*

*(run* OR hik* OR walk*)*

## Notes on search adaptation

Truncation symbols (*) were used to capture word variants (e.g., “run*” for run, running, runner).

Search syntax was adapted as required for each database interface.

No subject headings (e.g., MeSH, CINAHL Headings) were used due to variability in indexing and to maximise sensitivity across disciplines.

Search results from all databases were exported and combined for duplicate removal prior to screening.
